# Comparison of Thyroid Function Tests Among Type 2 Diabetes Patients With and Without Diabetic Nephropathy and Controls

**DOI:** 10.7759/cureus.70462

**Published:** 2024-09-29

**Authors:** J. T. V. Krishna Pavan Kumar, Uma M A

**Affiliations:** 1 General Medicine, PES Institute of Medical Sciences and Research, Kuppam, IND

**Keywords:** diabetes mellitus, diabetic nephropathy, hypothyroidism, thyroid dysfunction, thyroid function tests, thyroid screening

## Abstract

Background: Type 2 diabetes mellitus (T2DM) is a chronic metabolic disorder associated with hyperglycemia and impaired insulin function. Diabetic nephropathy (DN) is a common complication of T2DM, characterized by progressive kidney damage. Thyroid dysfunction, particularly hypothyroidism, has been increasingly recognised as a significant factor influencing the progression of DN.

Objective: To compare thyroid function tests in patients with T2DM with and without diabetic nephropathy and non-diabetic controls, and to assess the role of thyroid dysfunction in DN progression.

Methods: A hospital-based cross-sectional study was conducted among 225 participants. Patients were divided into three groups: T2DM with DN, T2DM without DN, and controls, with 75 participants in each group. Data collection included demographic details, comorbidities, and clinical assessments such as blood pressure, thyroid function tests, urinary albumin levels, glycated hemoglobin (HbA1c), renal function tests, serum electrolytes, and estimated glomerular filtration rate (eGFR). Statistical analyses were conducted using SPSS version 20.0 (IBM Corp., Armonk, NY, USA).

Results: The most common DN stage among T2DM patients was stage 4 (29, 38.7%), followed by stages 2 and 5 (19, 25.3% each). The T2DM with DN (47, 62.7%) group had a higher prevalence of overweight and obesity compared to the T2DM without DN group (38, 51.5%) and controls (24, 32%) (p value<0.001). Abnormal cardiovascular, respiratory, and abdominal examinations were most prevalent in the T2DM with DN group. Hypothyroidism was more prevalent in both T2DM groups (14, 18.7% each) compared to controls (2, 2.7%) (p value<0.004).

Conclusion: Our findings strengthen the evidence of an important link between T2DM and thyroid disorders. The high prevalence of thyroid dysfunction among T2DM patients highlights the critical need for routine screening and integrated management of both conditions to enhance patient outcomes.

## Introduction

Diabetes mellitus (DM) is a prevalent global health issue characterized by hyperglycemia, disrupted metabolism of proteins, carbohydrates, and lipids, pancreatic beta-cell dysfunction, and increased renal glucose reabsorption [1]. Among diabetic individuals, thyroid disorders are more common compared to the general population, with hypothyroidism being the most frequent [2]. The prevalence of thyroid dysfunction in diabetic patients ranges from 2.2% to 17%, with a higher incidence in women and a greater prevalence of hypothyroidism over thyrotoxicosis [3]. Imbalances in thyroid hormone levels can lead to significant metabolic disruptions, potentially exacerbating the risk of diabetic nephropathy (DN). Subclinical hypothyroidism has been identified as an independent risk factor for DN. Therefore, the study aims to explore the link between thyroid dysfunction and DN in type 2 diabetes mellitus (T2DM) patients.

Thyroid hormones play a crucial role in cellular metabolism and can act as antagonists to insulin. Imbalances in thyroid hormone levels can lead to significant metabolic disruptions, potentially exacerbating the risk of DN [4]. Subclinical hypothyroidism has been identified as an independent risk factor for DN [5], with serum thyroid-stimulating hormone (TSH) levels and tissue insulin sensitivity significantly impacting serum lipid profiles. Insufficient insulin sensitivity can magnify the effects of TSH variations, thereby increasing cardiovascular risk [6]. Undiagnosed thyroid dysfunction in diabetes patients can worsen pre-existing cardiovascular conditions and impede glycaemic control, underscoring the importance of timely diagnosis and management to improve metabolic control and overall health [7].

Thyroid hormones are integral to maintaining water and electrolyte balance, renal development, and renal transport functions. Thyroid dysfunction can adversely affect glomerular and tubular renal function, as well as influence the renin-angiotensin system and renal blood flow [8]. Globally, thyroid dysfunction is a prevalent endocrine disorder, with increased incidence among diabetics, particularly in the elderly and women [9,10]. Studies indicate that around 10% of individuals with diabetes experience thyroid disorders [11], with type 1 diabetics showing a higher prevalence compared to type 2 [12]. Recent research suggests that the frequency of thyroid disorders in type 2 diabetics has risen to levels comparable to those in type 1 diabetics [13].

Geographical variations and hereditary factors contribute to differences in diabetes manifestations and associated risks, such as thyroid dysfunction [14]. For instance, a cross-sectional study in Taiwan identified subclinical hypothyroidism as a significant risk factor for DN in T2DM patients [5], while research in southern China highlighted lower free triiodothyronine (FT3) levels in DN patients [15].

Therefore, the interplay between thyroid hormones and insulin in regulating metabolism and glucose homeostasis is well-established. Thyroid dysfunction can exacerbate diabetic complications, including DN, while DN can also influence thyroid function. Despite the growing body of evidence, the specific relationship between thyroid function and DN in T2DM remains unclear. To address this gap in literature the study aims to compare thyroid function tests in patients with T2DM with and without DN and non-diabetic controls.

## Materials and methods

Study design and setting

This hospital-based cross-sectional observational study was conducted among patients attending the Medicine Outpatient Department (OPD) at P.E.S. Institute of Medical Sciences and Research (PESIMSR), Kuppam, Andhra Pradesh. It was conducted over a period of 18 months, from January 2023 to June 2024.

Study participants

The study comprised three groups: T2DM patients with DN, T2DM patients without DN, and non-diabetic controls. The study included adults aged 25 years and above. Pregnant women, patients with type 1 DM, and those with a history of previous radiation exposure to the neck were excluded from the study. Patients were diagnosed with T2DM according to the American Diabetes Association criteria (glycated hemoglobin (HbA1C): ≥6.5% (48 mmol/mol), fasting blood sugar (FBS): ≥126 mg/dL (7.0 mmol/L)) [16]. For this study, an operational definition of diabetic nephropathy was used, focusing on incipient nephropathy (stage 2). The criteria for diabetic nephropathy included microalbuminuria ≥30 mg/g and an estimated glomerular filtration rate (eGFR) >30 mL/min/1.73m², as this operational approach emphasizes early detection and secondary prevention. By excluding patients with eGFR <30 mL/min/1.73m², we aimed to focus on individuals where screening and prevention strategies would have the greatest impact, thereby avoiding advanced kidney failure cases that would not benefit from early intervention [17]. The non-diabetic control group included participants who had never been diagnosed with DM and had no known thyroid conditions. Seventy-five participants were enrolled in each group by purposive sampling.

The sample size was calculated using OpenEpi software version 3.01 (www.OpenEpi.com). Based on the literature, the mean difference in FT3 levels of 0.5 pmol/L was observed between T2DM patients without DN and those with DN [18]. Assuming a 95% Confidence Interval (CI) and a sample size ratio of 1 (group 2/group 1), with a desired power of 90%, the initial estimate of the sample size was to be 55 participants per group. However, the final sample size was increased to 75 participants per group to account for potential dropouts.

Study procedure

Approval was obtained from the Research Monitoring Committee and the Institutional Ethics Committee (IEC) of PESIMSR, Kuppam, Andhra Pradesh. Eligibility of patients was determined through a review of their medical history and screening investigations, conducted only after obtaining written informed consent from the participants. A structured proforma was used to collect demographic data, personal history, and clinical examination details, including assessments of the cardiovascular, respiratory, and central nervous system (CNS) as well as abdominal examination. Body Mass Index (BMI) was calculated using the formula: body weight (in kilograms) divided by the square of height (in meters). Blood pressure (BP) measurements were taken in the sitting position after a rest period of more than 10 minutes. Three blood pressure readings were done for each participant, and the lowest of the three was taken. Fasting venous blood samples were collected for FBS and HbA1c analyses, and thyroid function was assessed using chemiluminescence immunoassay to confirm the levels of TSH, FT3, and free thyroxine (FT4). Urine specimens were collected from the first-morning urine sample, with patients instructed to avoid exercise for at least one hour before the examination. The urinary albumin-to-creatinine ratio was calculated. The dipstick test was used to assess proteinuria and eGFR was calculated using the Cockroft and Gault formula.

Statistical procedures

Data were entered into Microsoft Excel (Microsoft, Redmond, WA, USA), and statistical analysis was conducted using SPSS version 20.0 (IBM Corp., Armonk, NY, USA). Continuous variables were described as means with standard deviations, while categorical variables were presented as frequencies with percentages. The Chi-square test was used to compare categorical data, and the Student's t-test or one-way ANOVA was used for continuous data with a normal distribution. Pearson correlation analysis was used to analyze the relationships between renal indices and thyroid function. A p-value <0.05 was considered statistically significant.

## Results

**Figure 1:**
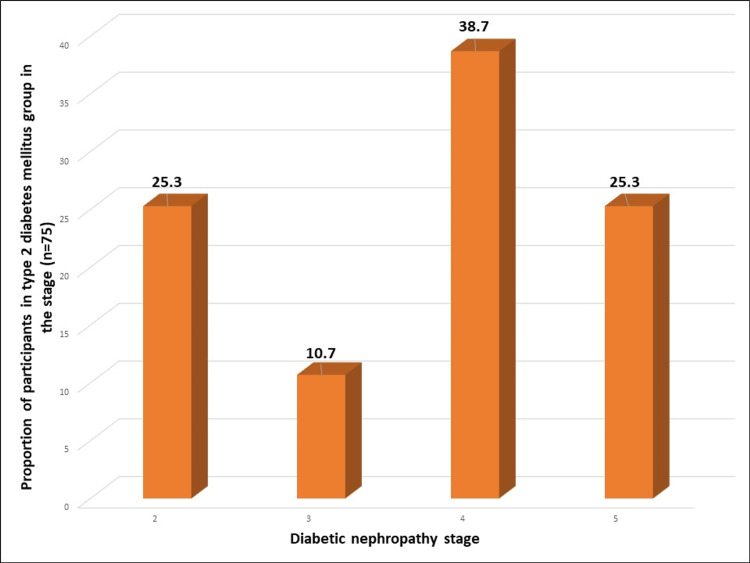
Distribution of diabetic nephropathy stage among the type 2 diabetes mellitus patients with diabetic nephropathy (n=75)

**Figure 2:**
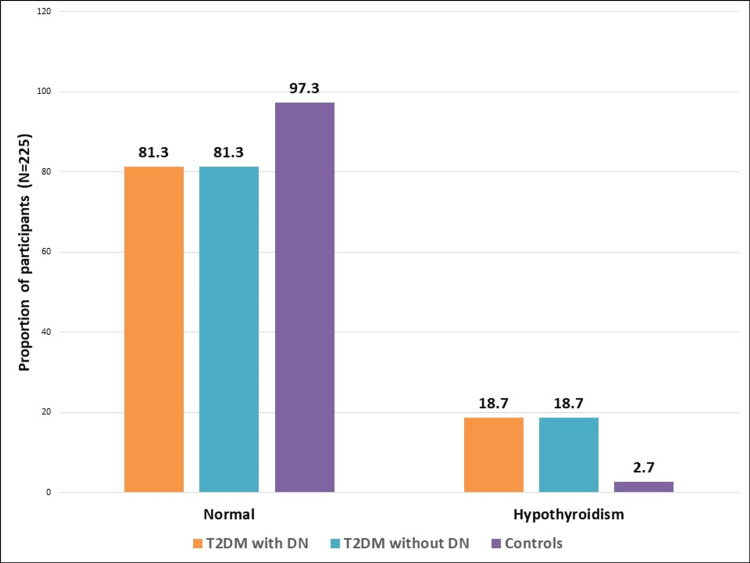
Comparison of thyroid function test between the groups (N=225) *statistically significant (p value=0.004) T2DM: Type 2 diabetes mellitus, DN: Diabetic nephropathy

A total of 225 participants were included in the study with 75 in each group. Most participants in the T2DM with DN and without DN groups were between 41-60 years, while younger individuals predominated in the control group. A higher proportion of males was observed in the diabetic groups compared to controls. Regarding BMI, both diabetic groups had a higher prevalence of overweight and obesity compared to controls. Patients with DN exhibited a higher frequency of abnormal physical examination findings, including heart sounds, respiration, abdomen, and jugular venous pressure (Table 1). Among participants in T2DM with DN group, the most prevalent DN stage was stage 4, affecting 38.7% (29) of participants, indicating moderate-to-severe kidney damage, while stages 2 and 5 were relatively evenly distributed (19, 25.3% each), and stage 3 was less common (8, 10.7%) (Figure 1). Hypothyroidism was more prevalent among both T2DM groups than controls (Figure 2).

**Table 1:** Socio-demographic and clinical characteristics of the participants in type 2 diabetes mellitus with and without diabetic nephropathy, and non-diabetic control groups (N=225) BMI: Body Mass Index p values are based on Chi-square tests, p-value <0.05 is considered significant

Characteristics	Type 2 diabetics with diabetic nephropathy n (%)	Type 2 diabetics without diabetic nephropathy n (%)	Controls n (%)	P value
Age (years)				
20-40	1 (1.3)	3 (4.0)	26 (34.7)	<0.001
41-60	38 (50.7)	38 (50.7)	35 (46.7)	
61-80	34 (45.3)	29 (38.7)	14 (18.6)	
81-100	2 (6.7)	5 (6.6)	0 (0.0)	
Gender				
Male	51 (68.0)	55 (73.3)	38 (50.7)	<0.001
Female	24 (32.0)	20 (26.7)	37 (49.3)	
BMI (kg/m^2^)				
Underweight	0 (0.0)	8 (10.8)	3 (4.0)	<0.001
Normal	28 (37.3)	28 (37.8)	48 (64.0)	
Overweight	34 (45.3)	26 (35.3)	24 (32.0)	
Obese	13 (17.4)	12 (16.2)	0 (0.0)	
Cardiovascular examination (heart sounds)				
Normal	60 (80.0)	65 (86.7)	70 (93.3)	0.05
Muffled	15 (20.0)	10 (13.3)	5 (6.7)	
Cardiovascular examination (murmur)				
No murmur	70 (93.3)	75 (100.0)	73 (97.3)	0.06
Murmur	5 (6.7)	0 (0.0)	2 (2.7)	
Respiratory Examination				
Normal	31 (41.3)	55 (73.3)	70 (93.3)	<0.001
Abnormal	44 (58.7)	20 (26.7)	5 (6.7)	
Abdominal Examination				
Normal	58 (77.3)	62 (82.7)	73 (97.3)	<0.001
Abnormal	17 (22.7)	13 (17.3)	2 (2.7)	
Central Nervous System Examination				
Normal	64 (85.3)	58 (77.3)	75 (100)	<0.001
Abnormal	11 (14.7)	17 (22.7)	0 (0.0)	
Jugular venous pressure				
Not elevated	64 (85.3)	75 (100)	72 (96.0)	<0.001
Elevated	11 (14.7)	0 (0.0)	3 (4.0)	
Thyroid function test				
Normal	61 (81.3)	61 (81.3)	73 (97.3)	0.004
Hypothyroidism	14 (18.7)	14 (18.7)	2 (2.7)	
Urine protein				
0	0 (0.0)	75 (100)	70 (93.3)	<0.001
1	34 (45.3)	0 (0.0)	4 (5.3)	
2	13 (17.3)	0 (0.0)	1 (1.3)	
3	28 (37.3)	0 (0.0)	0 (0.0)	
Urine sugar				
0	31 (41.3)	37 (49.3)	75 (100)	<0.001
1	29 (38.7)	15 (20.0)	0 (0.0)	
2	7 (9.3)	9 (12.0)	0 (0.0)	
3	8 (10.7)	14 (18.7)	0 (0.0)	

The renal function was significantly impaired in the DN group, as evidenced by lower eGFR and higher levels of serum creatinine and urea. All diabetic groups demonstrated significantly higher HbA1c and fasting blood sugar levels compared to controls. Despite the observed differences in other clinical parameters, there were no significant differences in TSH, FT3, or FT4 levels among the three groups (Table 2).

**Table 2:** Comparison of clinical characteristics of participants in type 2 diabetes mellitus with and without diabetic nephropathy, and non-diabetic control groups (N=225) BPM: Beats Per Minute, BP: Blood Pressure, RR: Respiratory Rate, TFT: Thyroid Function Test, TSH: Thyroid-Stimulating Hormone, FT3: Free Triiodothyronine, FT4: Free Thyroxine, HbA1c: Hemoglobin A1c, H_TLC: Total Leukocyte Count, H_Hb: Hemoglobin, H_PLT: Platelets, RFT: Renal Function Test, SE: Serum Electrolytes, Na+: Sodium, K+: Potassium, Cl-: Chloride, eGFR: Estimated Glomerular Filtration Rate. *P value obtained using ANOVA.

Clinical characteristics	Type 2 diabetes with diabetic nephropathy	Type 2 diabetes without diabetic nephropathy	Controls	P value*
	Mean (SD)	Mean (SD)	Mean (SD)	
Pulse (BPM)	87.8 (17.1)	89.8 (9.6)	66.3 (17.3)	<0.001
Systolic Blood Pressure (mmHg)	139.4 (31.2)	128.9 (22.8)	126.5 (13.6)	<0.001
Diastolic Blood Pressure (mmHg)	81.5 (16.8)	78.2 (12.6)	84.3 (7.6)	<0.001
RR (CPM)	23.9 (5.1)	19.4 (3.2)	14.4 (1.9)	<0.001
TFT (TSH)	3.2 (1.7)	3.2 (1.5)	2.2 (1.4)	0.13
FT3	3.7 (1.4)	4.7 (1.6)	7.2 (1.2)	0.08
FT4	17.2 (4.7)	17.2 (3.8)	18.9 (5.0)	0.06
HBA1c (%)	9.5 (1.5)	9.2 (2.3)	3.7 (1.5)	<0.001
Fasting sugar (mg/dL)	182.9 (42.5)	182.5 (55.3)	94.8 (7.4)	<0.001
H_TLC (thousand per microlitre)	9.6 (3.3)	8.9 (3.3)	7.5 (1.9)	<0.001
H_Hb (%)	9.6 (2.3)	10.3 (1.7)	11.8 (1.7)	0.01
H_PLT (Thousand)	243.3 (64.3)	242.4 (68.5)	290.4 (86.4)	0.02
RFT_Urea (mg/dl)	79.2 (47.9)	23.6 (11.3)	30.9 (10.5)	<0.001
RFT_Creatinine (mg/dl)	4.2 (2.3)	1.1 (0.2)	0.7 (0.4)	<0.001
SE_Na+ (mmol/L)	138.2 (10.1)	134.9 (5.9)	134.6 (2.9)	<0.001
SE_K+ (mmol/L)	4.6 (1.2)	4.0 (0.6)	4.5 (0.5)	<0.001
SE_Cl- (mmol/L)	104.3 (9.3)	101.6 (5.9)	100.7 (3.2)	<0.001
eGFR (ml/min)	31.1 (21.7)	77.7 (19.9)	114.9 (14.5)	<0.001

In those with DN, TSH had a weak, non-significant positive correlation with serum creatinine (r = 0.21, p = 0.07) and serum urea (r = 0.05, p = 0.62), while it was negatively correlated with eGFR (r = -0.16, p = 0.14), though not statistically significant. In contrast, patients without DN showed no significant correlation between TSH and serum creatinine (r = -0.01, p = 0.89) or serum urea (r = -0.07, p = 0.54), but a significant positive correlation with eGFR (r = 0.26, p = 0.02) (Table 3).

**Table 3:** Correlation between thyroid function and related renal indexes of type II diabetes mellitus patients with and without diabetic nephropathy (N=150) TSH: Thyroid-Stimulating Hormone, eGFR: Estimated Glomerular Filtration Rate. r denotes the Pearson correlation coefficient, p value < 0.05 is considered significant.

Clinical Characteristics	TSH			
	Type II diabetes with diabetic nephropathy		Type II diabetes without diabetic nephropathy	
	r value	p-value	r value	p-value
Serum Creatinine	0.21	0.07	-0.01	0.89
Serum Urea	0.05	0.62	-0.07	0.54
eGFR	-0.16	0.14	0.26	0.02

## Discussion

The study aimed to compare thyroid function tests in patients with T2DM mellitus with and without DN and non-diabetic controls. A higher prevalence of overweight and obesity was observed in T2DM patients with DN, consistent with findings from Chen et al. and Ravindran et al. in India [19,20]. Obesity, associated with chronic low-grade inflammation, adipokine dysregulation, insulin resistance, and comorbidities like hypertension and dyslipidemia, contributes to the pathogenesis and progression of DN through oxidative stress, endothelial dysfunction, and proinflammatory pathways [21,22]. Maintaining a healthy BMI in controls suggests a protective effect against T2DM, highlighting the importance of early weight management interventions in T2DM patients.

This study highlights the significantly worse cardiovascular and respiratory profiles in patients with DN compared to those with T2DM without DN and controls. Patients with DN exhibited higher pulse rates, systolic blood pressure (SBP), diastolic blood pressure (DBP), and respiratory rates, reflecting the increased cardiovascular burden associated with DN aligning with previous findings linking chronic hyperglycemia, hypertension, and inflammation to cardiovascular risks like heart attacks and strokes [23,24]. A notably higher prevalence of muffled heart sounds and cardiac murmurs in the DN group further emphasizes the potential cardiac complications. The study also identified a higher prevalence of abnormal respiratory findings in the DN group, consistent with prior research showing impaired pulmonary function in T2DM patients with DN [25]. Additionally, a significant proportion of DN patients exhibited abnormal abdominal findings and CNS abnormalities. This underscores the importance of comprehensive management strategies for T2DM patients with DN to address cardiovascular, respiratory, abdominal, and cognitive complications.

This study highlights the prevalence of thyroid dysfunction, that is hypothyroidism was significantly higher in those with T2DM compared to controls. These findings align with existing research indicating an elevated risk of thyroid dysfunction in diabetic populations [26]. Chao et al.'s cross-sectional study in China found elevated TSH and reduced FT3 and FT4 levels in DN patients, suggesting a link between thyroid hormone levels and DN [27]. Similarly, Zhao et al. observed higher TSH and lower FT3 in DN patients, indicating a potential connection between thyroid dysfunction and DN [18]. In alignment with these studies, our research also found lower FT3 levels in DN patients, with TSH levels comparable across both T2DM groups but higher than those of controls. These results reinforce the potential role of thyroid dysfunction in the pathophysiology of diabetic nephropathy and warrant further exploration into its clinical implications.

Iwakura et al. identified a negative correlation between TSH and eGFR (p value<0.05), implying that thyroid dysfunction may influence DN progression, although no direct relationship with diabetes control or renal decline was reported [26]. In contrast, our study revealed a statistically significant positive correlation between TSH and eGFR, but only in the T2DM group without DN, not in those with DN. This discrepancy may indicate differing thyroid-renal dynamics at various stages of DN. Additionally, Zou et al. and Liu et al. showed that higher FT3 levels might have a protective effect against the development and progression of DN in T2DM patients [27,28]. These findings highlight the potential role of thyroid function in influencing renal outcomes in T2DM Incorporating thyroid function tests into regular clinical assessments could lead to earlier detection of thyroid dysfunction and help guide interventions that may improve outcomes in this high-risk population.

The comparison of urine protein levels across different groups highlights the significant role of proteinuria in assessing renal function and DN progression. This supports the existing literature that emphasizes proteinuria as a key indicator of renal dysfunction and a predictor of adverse outcomes, such as end-stage renal disease [29]. In contrast, T2DM without DN had no detectable urine protein, suggesting preserved renal function. The control group mostly had no proteinuria, with a small fraction showing mild levels, indicating the necessity to consider other potential causes of kidney damage beyond diabetes. Recent studies have also emphasized the potential of novel biomarkers and emerging categories for earlier detection of DN, such as tubular markers and markers associated with inflammation and oxidative stress, suggesting the need for further research [30].

Elevated HbA1c and fasting blood sugar levels in DN patients reflect poor glycaemic control and its adverse effects on kidney function. Increased urine sugar levels in these patients further stress the need for effective glycaemic management. The predominance of advanced DN stages in our cohort indicates the progressive nature of the disease, emphasizing the need for early intervention. Additionally, electrolyte imbalances and altered hematological parameters in DN patients highlight the importance of comprehensive monitoring and management. Future research should focus on integrating biomarker assessments, exploring novel markers for earlier detection, and evaluating the impact of lifestyle modifications.

The study's strengths include a comprehensive assessment of various clinical parameters, robust statistical analysis, and a large sample size of 225 participants enhancing the results' reliability. However, the limitations need to be addressed. These include the cross-sectional design, which restricts causal inference, and its single-center approach, which may limit generalizability. Additionally, there is potential selection bias from the specific patient population and a lack of control for confounding factors such as medication use and other comorbidities.

## Conclusions

In our study, we observed that thyroid dysfunction (hypothyroidism) is significantly more prevalent in T2DM patients compared to controls, with lower FT3 levels observed in patients with diabetic nephropathy. These findings indicate that thyroid function may play a role in the progression of diabetic nephropathy. Incorporating thyroid function tests into routine assessments for T2DM patients, especially those at risk of DN, could improve patient outcomes by facilitating earlier diagnosis and targeted management of thyroid-related complications. Further research is needed to explore the complex interactions between thyroid function and renal health in diabetic populations.
